# Comparative Study of a Modified Sub-Tenon’s Capsule Injection of Triamcinolone Acetonide and the Intravenous Infusion of Umbilical Cord Mesenchymal Stem Cells in Retinitis Pigmentosa Combined With Macular Edema

**DOI:** 10.3389/fphar.2021.694225

**Published:** 2021-09-27

**Authors:** Tongtao Zhao, Hongxuan Lie, Fang Wang, Yong Liu, Xiaohong Meng, Zhengqin Yin, Shiying Li

**Affiliations:** ^1^ Southwest Hospital, Third Military Medical University (Army Medical University), Chongqing, China; ^2^ Key Lab of Visual Damage and Regeneration & Restoration of Chongqing, Chongqing, China; ^3^ Changhai Hospital, The Second Military Medical University (Naval Medical University), Shanghai, China

**Keywords:** umbilical cord mesenchymal stem cells, triamcinolone acetonide, retinitis pigmentosa, macular edema, sub-Tenon’s capsule injection

## Abstract

Retinitis pigmentosa (RP) is a hereditary retinal degenerative disease leading to eventual blindness. When RP is combined with macular edema (ME), the visual impairment further worsens. We compared a modified sub-Tenon’s capsule injection of triamcinolone acetonide (TA) and the intravenous infusion of umbilical cord mesenchymal stem cells (UCMSCs) in the treatment of RP combined with ME (RP-ME) to assess their safety and efficacy in eliminating ME and restoring visual function. A phase I/II clinical trial enrolled 20 patients was conducted. All patients were followed up for 6 months. There were no severe adverse effects in both groups. In retinal morphological tests, the central macular thickness (CMT) in TA group significantly decreased at first week, first and second month after injection (*p* < 0.05). The CMT in UCMSCs group significantly decreased at first month after infusion. The rate of reduction of CMT in TA group was significantly greater than that in UCMSCs group at second month (*p* < 0.05). Reversely, the rate of reduction of CMT in UCMSCs group was significantly greater than that in TA group at sixth month (*p* < 0.05). In visual functional test, although there were no significant differences in visual acuity or visual fields within each group or between groups, but the amplitude of P2 wave of flash visual evoked potential (FVEP) showed significant increasing in TA group at second month in UCMSCs group at sixth month (*p* < 0.05). At 6th month, the rate of growth in the amplitude of P2 wave in USMCSs group was significantly greater than that in TA group (*p* < 0.05). This study suggests both modified sub-Tenon’s capsule injection of TA and intravenous infusion of UCMSCs are safe for RP-ME patients. TA injection is more effective at alleviating ME while improving visual function in a short term. UCMSC intravenous infusion shows slow but persistent action in alleviating ME, and can improve the visual function for a longer time. These approaches can be applied separately or jointly depending on the disease condition for patients to benefit maximumly.

**Clinical Trial Registration:** http://www.chictr.org.cn, identifier ChiCTR-ONC-16008839

## Introduction

Retinitis pigmentosa (RP) is a group of hereditary retinal degenerative diseases characterized by progressive RPE dysfunction and photoreceptor loss. The clinical symptoms include poor night vision, visual field constriction and eventual blindness (Anasagasti et al., 2012). To date, there are no effective interventions to prevent this disease from advancing. When RP is combined with macular edema (ME), the condition is more difficult to treat, and the visual impairment becomes worse (Strong et al., 2017; Tan et al., 2021). The prevalence of ME in RP ranges from 11 to 49% depending on different approaches used for examination (Hajali et al., 2008; Huckfeldt and Comander, 2017; Salvatore et al., 2013). Thus, for patients who have RP combined with ME (RP-ME), effective control of the ME is crucial in order to rescue visual function. The mechanisms involved in RP-ME may include breakdown of the blood-retinal barrier, retinal pigment epithelial and Müller cell dysfunction, production of antiretinal antibodies, etc (Strong et al., 2017). Inflammation and auto-immune processes were found to be the major underlying pathogenesis (Yoshida et al., 2013; Narayan et al., 2016; Strong et al., 2017). Given the complicated mechanisms RP-ME has, a therapeutic regime covering multiple perspectives of action is needed.

Cortical steroids are important therapeutic agents for tissue inflammation and edema, among which, triamcinolone acetonide (TA) is widely used because of its long-acting anti-inflammation and immune modulation effects (Saraiva et al., 2003). Local administrations including intravitreal injection and sub-Tenon’s capsule injection of TA have been applied in many retinal pathological conditions combining with ME (Ip et al., 2004). Compared with intravitreal injection, the sub-Tenon’s capsule injection has less risks and complications. For RP-ME, a short-term study has demonstrated a beneficial effect of sub-Tenon’s capsule injection of TA (Karasu, 2020). To our knowledge, there are still no studies focusing on the long-term observation and visual functional assessments following sub-Tenon’s capsule injection of TA in RP-ME.

On the other hand, the attempt of stem cell therapy in RP has been long explored. As a major kind of mesenchymal stem cells, umbilical cord mesenchymal stem cells (UCMSCs) have a wide range of biological effects, such as anti-inflammation, immune modulation, paracrine and neurotrophy (Nagamura-Inoue & He, 2014; Xiuying et al., 2014; Zou et al., 2012). Many clinical trials have demonstrated beneficial effects of intravenous infusion of UCMSCs in the treatment of different systemic diseases including neurological, cardiac and osteoarticular disorders (Bartolucci et al., 2017; Mukai et al., 2018; Riordan et al., 2018). In our previous study, UCMSCs were intravenously administered in RP patients. During the 12 months of follow up, most patients improved their visual acuities in the first 3 months, and maintained their visual function for the whole duration of follow up. Besides, the visual acuity related life quality, which was assessed by the relevant questionnaire scores, was significantly increased during first 3 months (Zhao et al., 2020). However, it is still unknown if UCMSCs can help alleviate the RP-ME. Although UCMSCs and TA have some common functions, their effectiveness may vary in onset time, maintaining duration or magnitude of improvement. To know the differences between these two agents and make single or combined treatment regime accordingly will help patients achieve maximum benefit. In this study, we compared the safety and efficacy of an intravenous infusion of UCMSCs and a modified sub-Tenon’s capsule administration of TA in RP-ME. Here, we report the results of our study.

## Materials and Methods

### Study Design

**FIGURE 1:**
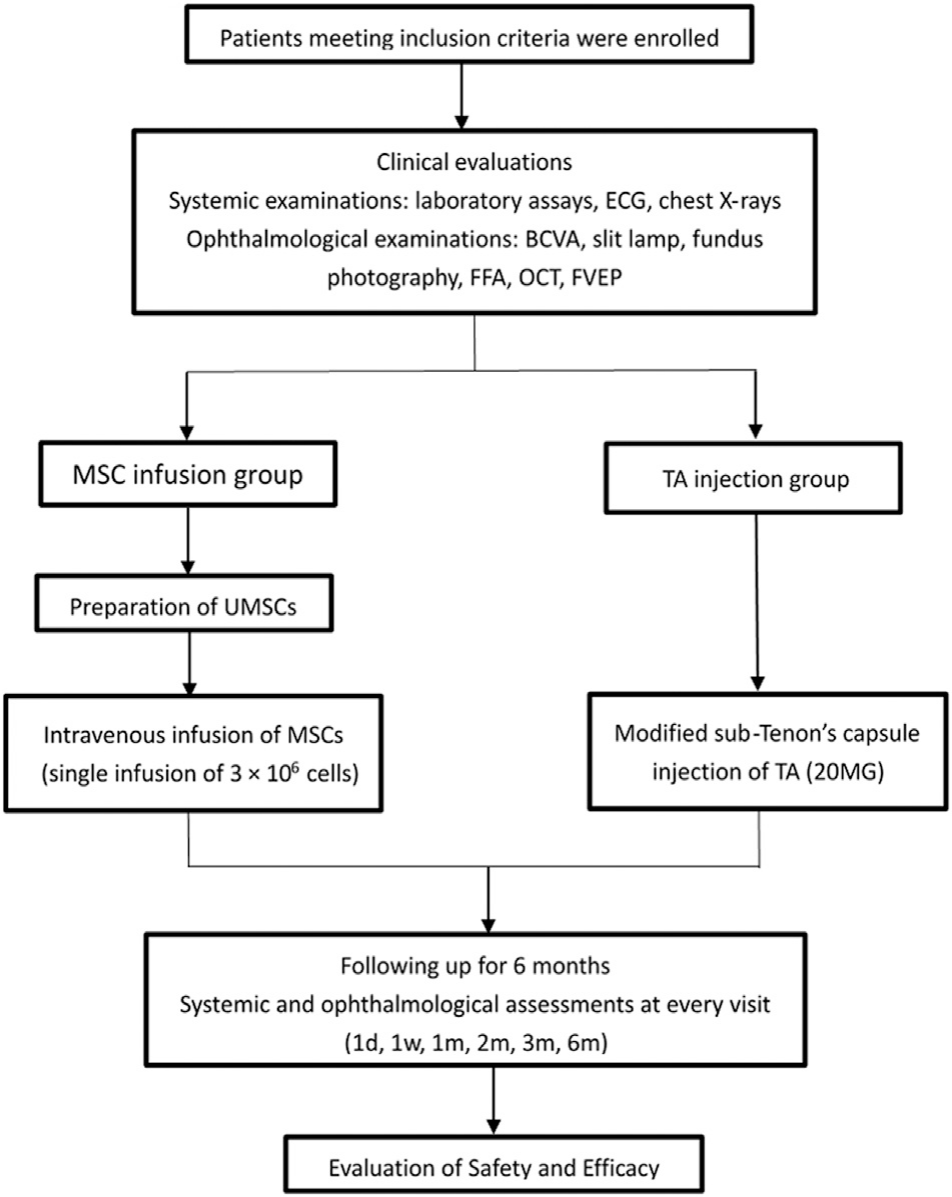
Flow chart of the study.

This is a prospective, open label, randomized, phase I/II clinical trial. This study was approved by the Medical Ethics Committee of Southwest Hospital, the Army Medical University, and conducted between July 2016 and March 2018. The subjects in the UCMSCs infusion group received a single intravenous infusion of 3 × 10^6^ UCMSCs, and the subjects in the TA injection group received a single injection of 20 mg of TA. All of the subjects were followed for 6 months. Systemic and ophthalmological examinations were performed to assess the safety and efficacy (Figure 1). The study adhered to the principles of the Declaration of Helsinki and the International Ethical Guidelines for Biomedical Research Involving Human Subjects and was registered in the Chinese Clinical Trial Registry (Primary Registry of the International Clinical Trials Registry Platform of the World Health Organization) (ChiCTR-ONC-16008,839). Every patient that was recruited for the study signed a written informed consent form.

### Umbilical Cord Mesenchymal Stem Cells Preparation

The UCMSCs used for this study were derived from neonatal umbilical cord tissue according to the standard protocol and met the criteria approved by the International Society for Cellular Therapy (Dominici et al., 2006; Majore et al., 2011; Mushahary et al., 2018; Salehinejad et al., 2012). The preparation of the cells was performed by the Biotherapy Centre of the Army Medical University. Briefly, the Wharton’s Jelly tissue was aseptically cut into a homogenate of 2–3 mm^3^ tissue blocks, then the blocks were seeded into T75 flasks in Mesenchymal Stem Cell Basal Medium (DAKEWE, Beijing, China) supplemented with 5% UltraGROTM (HPCFDCRL50, Helios). The tissue blocks were cultured at 37°C, 5% CO2 for about 10 days for UCMSCs to reach confluence. Then cells were digested with 0.125% Trypsin and passaged at 1:3 ratio. Each enzymatic digestion step was considered to be a passage. Cells at P3-P5 were used for infusion. All infused UCMSCs were prepared based on the criteria approved by the International Society for Cellular Therapy. The final products met all of the following criteria: cell viability was no less than 95%; the cells were sterile; the cells did not have endotoxins, *mycoplasma*, hepatitis B, hepatitis C, or syphilis; and the cells exhibited expression of the appropriate surface markers (the positive rate of CD34 and CD45 was less than 0.5%, the positive rate of CD29 was more than 95%, the positive rate of CD90 was more than 95%, the positive rate of CD105 was more than 95%, and the positive rates of CD71 and CD73 were more than 95%). (Supplementary Figures S1–S3).

### Patient Screening

The inclusion criteria were as follows:

1) Patients aged 18–65 years (including 18 and 65 years) who had signed an informed consent form.
2) Patients with at least one eye or both eyes suffering from impaired vision caused by retinitis pigmentosa combined with macula edema.
3) Patients who voluntarily selected UCMSCs infusion or TA injection for the treatment of retinitis pigmentosa combined with macula edema.
4) Using the Early Treatment Diabetic Retinopathy Study (ETDRS) visual acuity checklist at a distance of 4 m, the best corrected visual acuity scores was ≥19 letters and ≤73 letters (or the equivalent of about Snellen eyesight from 20/400 to 20/40).
5) Patients who had the ability to adhere to the study follow-up and protocol requirements.

The exclusion criteria were as follows:

1) Patients with any active intraocular inflammation, infection, or concomitant diseases in their eyes that may affect the interpretation of the results of the study or may lead to visual impairment, including severe cataracts, glaucoma, retinal vascular obstruction, retinal detachments, macular holes, vitreous macular traction, and choroidal neovascularization.
2) Patients with a history of intraocular surgery.
3) Patients with a stroke, coronary heart disease, renal insufficiency requiring dialysis or kidney transplantation, or other systemic chronic diseases.
4) Patients with hypertension (systolic pressure >140 mmHg or diastolic pressure >90 mmHg) or diabetes that cannot be controlled by drugs.
5) Females who planned to become pregnant within the next 6 months, were pregnant or were lactating.

### Intravenous Infusion of Umbilical Cord Mesenchymal Stem Cells

The vital signs of all of the patients involved in this study, including their temperature, respiration rate, pulse, blood pressure, oxygen saturation, electrocardiogram signals and pain severity, were continuously monitored before, during and up to 2 h after the infusion. The patients underwent treatment only when all of their vital signs were normal. Every patient received a sequential intravenous infusion of UCMSCs (3 × 106 cells, 250 ml per person) through the dorsal hand vein within 60 min. The infusion was stopped immediately and treated in a timely manner when immune rejection, anaphylaxis, and infusion reactions, such as headache, dizziness, nausea and vomiting, occurred.

### Modified Sub-tenon’s Capsule Injection of Triamcinolone Acetonide

A system of modified sub-Tenon’s capsule injection was developed (Patent No. ZL 2013 20740,202.0, China). Briefly, a 2 mm incision was made 10 mm to the limbus at the superotemporal bulbar conjunctiva. The conjunctiva, bulbar fascia and Tenon’s capsule were bluntly dissected exposing the sclera. A specially developed curved needle with a blunt tip was inserted through the incision and posteriorly run along the surface of the sclera until it reached the posterior pole that corresponded to the macular area. Then, 20 mg of TA (LISAPHARMA, Italy) was injected into the sub-Tenon’s capsule space. The needle was slowly withdrawn to avoid any leaking of the drug, and pressure was applied to the conjunctiva incision for a few seconds. No sutures were needed. An antibiotic ointment was applied in the injected eye, and the eye was patched for 1 day. Antibiotic eye drops were used for a few days following the injection.

### Clinical Evaluation

The safety and efficacy parameters were evaluated at baseline and at first day, first week, and first, second, third, sixth month after the intravenous UCMSCs infusion or the TA injection. Relevant blood biochemical indexes were measured before and after treatment. The best corrected visual acuity (BCVA) was used as the standard for visual acuity evaluation and was determined by the Early Treatment Diabetic Retinopathy Study (ETDRS) alphabet (Topcon CC 100 XP, Japan). Optical computed tomography (OCT) scans were performed to evaluate the central macular thickness (CMT) (OCT-1000, Topcon, Japan). The rate of change (ROC) of CMT at every visit was calculated as (%): (value measured - baseline value)*100/baseline value. The visual fields were tested using a Humphrey Visual Field Analyzer. The sum of visual field sensitivity (dB) was calculated. The flash visual evoked potential (FVEP) was tested according to the standardized procedures developed by the International Society for Clinical Electrophysiology of Vision (ISCEV) (Espion E2 Diagnosis, U.S.A.). The ROC of amplitude of P2 wave was calculated as (%): (value measured - baseline value)*100/baseline value.

### Statistical Analysis

The IBM SPSS Statistics 26 (IBM, Corp, Armonk, NY, United States) software was used to describe and analyze the data. Continuous variables were described by mean ± SD. The independent-samples *t*-test or the Mann–Whitney U test was used for comparing continuous variables between two groups. The Wilcoxon signed-rank test was used for detecting differences between variables before and after intervention. *p* < 0.05 was considered statistically significant.

## Results

### Safety Assessments of Triamcinolone Acetonide Injection and Umbilical Cord Mesenchymal Stem Cells Infusion

**FIGURE 2:**
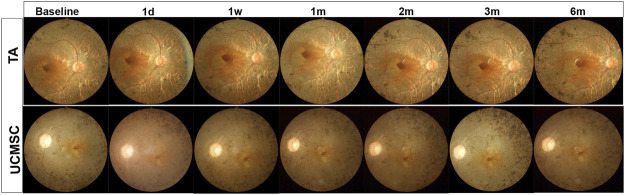
Fundus photograph of representative RP patient in both groups. The fundus photograph shows typical fundus appearance of RP, with yellowish color of optic disc, thin blood vessels, gray retinal color and osteocyte like pigment deposits in peripheral retina. There were no adverse effects such as hemorrhage, exudation or inflammatory signs found at all follow ups in both groups.

There were 20 patients (40 eyes) enrolled in the study, and they were randomized into TA injection group and UCMSCs infusion group (Table 1). All of the patients were clinically diagnosed with RP-ME. In the TA injection group, there were no local or systemic adverse effects for all of the patients before or after the injection. In the UCMSCs group, the vital signs of all patients were stable during the infusion process. There were no adverse effects such as fever, infection, headache, vertigo, nausea, vomiting, allergic reactions or immune rejection reactions that happened during or after the UCMSCs infusion. There were no hemorrhage, exudation or inflammatory signs found during all follow ups in both groups (Figure 2). There were three patients who had a substantial increase of interleukin-6 (IL-6) in UCMSCs group at first week, and then the IL-6 decreased to normal level subsequently. No definite IL-6 change was found in TA group. There were no significant changes in white blood cell, liver and renal function, blood glucose, C-reactive protein (CRP), procalcitonin (PCT) throughout the follow-up period in both groups (Supplementary Tables S1, S2).

**TABLE 1:** Baseline assessments of the subjects.

	TA	MSC	*p* Value
Subjects (eyes)	10 (20)	10 (20)	
Sex (Male/Female)	8/2	6/4	
Age (years)	45.6 ± 12.2	38.3 ± 13.5	>0.05^a^
Central Macular Thickness (μm)	330 ± 42.63	294.13 ± 29.11	>0.05^b^
BCVA (ET^a^S numbers)	56.0 ± 13.8	57.4 ± 20.3	>0.05^a^
visual field sensitivity (dB)	494.80 ± 114.53	538.56 ± 176.62	>0.05^b^
Amplitude of P2 in ^a^EP (μv)	8.38 ± 4.49	12.18 ± 7.69	>0.05^b^
Latency of P2 in FV^a^ (ms)	111.33 ± 12.17	109.88 ± 11.16	>0.05^b^

a independent *t* test^a^

b Mann–Whitney *U* test.

### Assessments of Central Macular Thickness

#### Central Macular Thickness Analysis in Each Group

**FIGURE 3:**
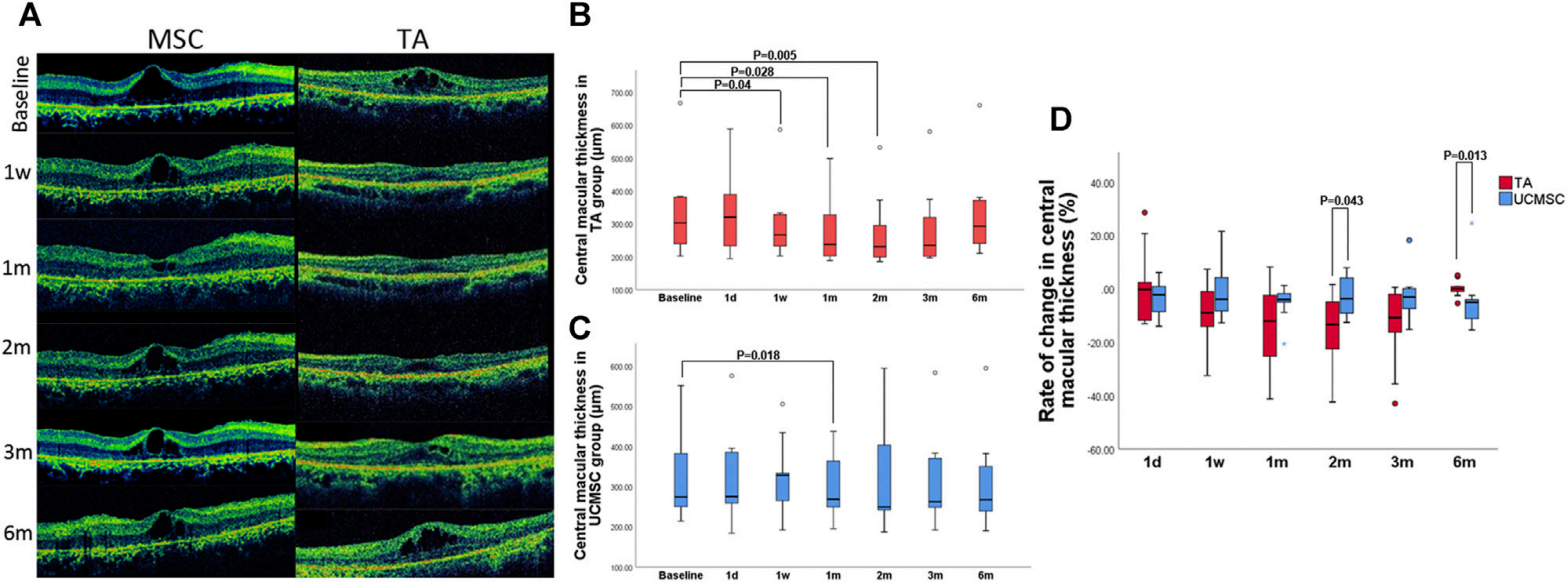
Assessment of central macular thickness. **(A)** Morphological changes in the UCMSCs infusion and TA injection groups **(B)** In the TA group, the CMT significantly decreased at first week, first and second month (*p* = 0.04,0.028,0.005 respectively, *n* = 20, Wilcoxon signed-rank test). At 3rd and sixth month, the CMT gradually increased to baseline level (*p* > 0.05, *n* = 20, Wilcoxon signed-rank test). **(C)** In the UCMSCs group, the CMT at first month was significantly lower than baseline (*p* = 0.018, *n* = 20, Wilcoxon signed-rank test). The CMT at third and sixth month were also lower than baseline, but there was no significant difference (*p* > 0.05, *n* = 20, Wilcoxon signed-rank test). **(D)** Comparison of the rate of change in CMT between two groups: At 2nd month, the rate of reduction of CMT in TA group was significantly greater than that in UCMSCs group (*p* = 0.043, *n* = 20, Mann–Whitney U test). At 6th month, the rate of reduction of CMT in UCMSCs group was significantly greater than that in TA group (*p* = 0.013, *n* = 20, Mann–Whitney U test).

There was no significant difference between the baselines of the two groups (*p* > 0.05, *n* = 20, Mann–Whitney U test). Compared with baseline, CMT in TA group significantly decreased at first week, first and second month (*p* = 0.04,0.028,0.005 respectively, *n* = 20, Wilcoxon signed-rank test). There was a rebound at third and sixth month when the CMT increased again, and was not statistically different from baseline (*p* > 0.05, *n* = 20, Wilcoxon signed-rank test) (Figures 3A,B). In the UCMSCs infusion group, CMT at first month was significantly lower than baseline (*p* = 0.018, *n* = 20, Wilcoxon signed-rank test). The CMT at third and sixth were also lower than baseline, but there was no significant difference (*p* > 0.05, *n* = 20, Wilcoxon signed-rank test) (Figures 3A,C).

#### Comparison of Rate of Change in Central Macular Thickness

To further evaluate the effect of both agents on reducing macular edema, the rate of change (ROC) in CMT was calculated. In TA group, the ROC was more negative at first and second month, then it was gradually close to 0 at third and sixth month. In MSC group, the ROC showed a relatively small amplitude of variation. When compared with UCMSCs group, the ROC of TA group was significant different (more negative) at second month indicating a significant decrease in CMT (*p* = 0.043, n = 20, Mann–Whitney U test). Whereas, a reverse change was observed at sixth month, when the ROC of UCMSCs group was significantly lower (more negative) than that in TA group (*p* = 0.013, n = 20, Mann–Whitney U test), indicating a stronger act of UCMSCs in reducing CMT (Figure 3D).

### Assessments of Visual Functions

#### Comparison of Visual Field Sensitivity and BCVA

**FIGURE 4:**
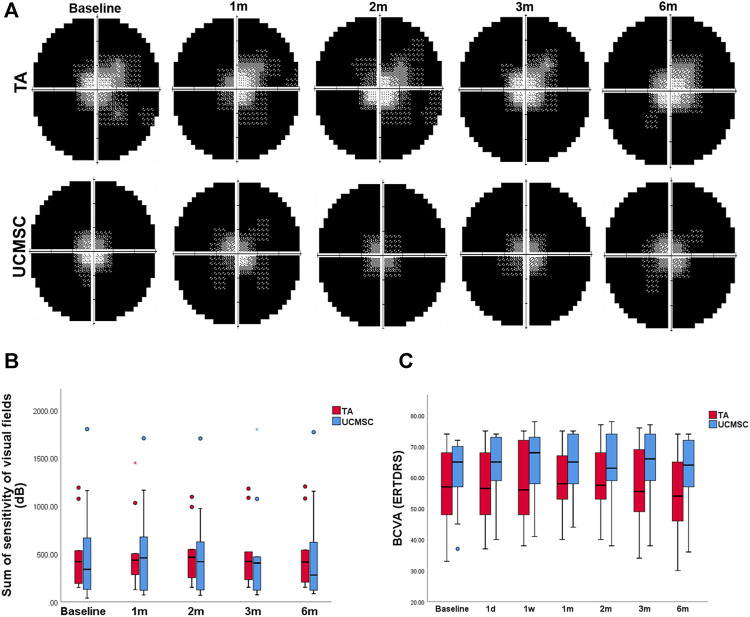
Assessments of BCVA and visual field sensitivity. **(A, B)** The sum of visual field sensitivity in TA group peaked at the second month without a significant difference (*p* > 0.05, *n* = 20, Wilcoxon signed-rank test), and then decreased to the baseline level at the sixth month. In the UCMSCs infusion group, this value increased at first, second and third month without significant differences (*p* > 0.05, *n* = 20, Wilcoxon signed-rank test). Then it decreased to baseline value at sixth month. There were no significant differences found within each group or between the two groups (*p* > 0.05, *n* = 20, Mann–Whitney U test). **(C)** There were no significant differences of BCVA found within each group or between the two groups (*p* > 0.05, *n* = 20, Wilcoxon signed-rank test and Mann–Whitney U test).

The total value of visual field sensitivity was calculated. In the TA injection group, this value peaked at the second month without a significant difference (*p* > 0.05, *n* = 20, Wilcoxon signed-rank test). In the UCMSCs group, the overall sensitivity value increased at first, second and third month, but was not statistically different (*p* > 0.05, *n* = 20, Wilcoxon signed-rank test). When comparing the visual field sensitivities of the two groups, there were no significant difference found between any follow ups (*p* > 0.05, *n* = 20, Mann–Whitney U test) (Figures 4A,B). Compared with baseline, the BCVA showed no significant differences at any follow up in each group (*p* > 0.05, *n* = 20, Wilcoxon signed-rank test). The intergroup comparison found no significant difference neither (*p* > 0.05, *n* = 20, Mann–Whitney U test) (Figure 4C).

#### Comparison of Flash Visual Evoked Potential

**FIGURE 5:**
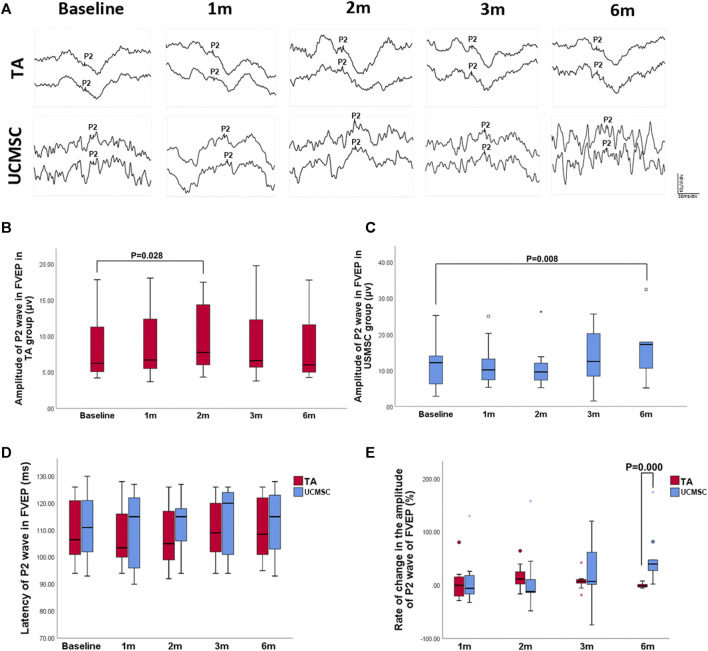
Assessments of FVEP. **(A, B)** In the TA injection group, the amplitude of P2 wave in FVEP significantly increased at second month (*p* = 0.028, *n* = 20, Wilcoxon signed-rank test). **(A, C)** In UCMSCs infusion group, the amplitude of P2 wave showed a significantly increase at sixth month (*p* = 0.008, *n* = 20, Wilcoxon signed-rank test). **(D)** There were no significant differences of latency of P2 wave among follow ups within each group or between the two groups (*p* > 0.05). **(E)** The ROC in amplitude of P2 wave in UCMSCs group was significantly higher than that of TA group at sixth month (*p* = 0.000, *n* = 20, Mann–Whitney U test).

To obtain stable parameters, each waveform of FVEP was the result of overlay of 64 times of detections, and two paralleling waveforms were generated for each eye (Figure 5A). In the TA injection group, the amplitude of P2 wave in FVEP significantly increased at second month (*p* = 0.028, *n* = 20, Wilcoxon signed-rank test). At 3rd and sixth month, it dropped down and had no statistical differences with baseline level (*p* > 0.05) (Figures 5A,B). In UCMSCs infusion group, the amplitude of P2 wave demonstrated a trend of increase at third and sixth month, but not until sixth month did it show a significant rise comparing with baseline (*p* = 0.008, *n* = 20, Wilcoxon signed-rank test) (Figures 5A,C). For the latency of P2 wave, there were no significant differences among follow ups within each group or between the two groups (*p* > 0.05) (Figure 5D). To compare the two group more precisely, the ROC of amplitude of P2 wave was also calculated. The ROC in amplitude of P2 wave in UCMSCs group was significantly higher than that of TA group at sixth month (*p* = 0.000, *n* = 20, Mann–Whitney U test) (Figure 5E).

## Discussion

As a major worldwide retinal degenerative disease that causes blindness, the hereditary modes of RP can be autosomal dominant (30–40%), autosomal recessive (50–60%), or a X-linked trait (5–15%) (Hartong et al., 2006). The inherited nature of RP leads to progressive photoreceptor apoptosis and irreversible visual loss. However, when RP is combined with ME, the impairment of visual function becomes worse. The impairment of the blood retinal barrier (BRB) is thought to be the main cause of ME in RP (Vinores et al., 1995; Larsen et al., 1997). With the progression of RP, both retinal vascular endothelium and RPE lose their normal intercellular junctions, which gives rise to increased retinal vascular permeability and a flow of interstitial fluid from the choroid to the retinal tissue (Larsen et al., 1997; Marmor, 1999; Vinores et al., 1999). Inflammation and auto-immune processes play an important role in the pathogenesis of vascular endothelium and RPE dysfunction and the subsequent breakdown of the BRB(Yoshida et al., 2013; Narayan et al., 2016; Strong et al., 2017).

Different methods have been used to treat RP-ME, such as systemic administration of carbonic anhydrase inhibitors or steroids, intravitreal injection of steroids or anti-VEGF agents, laser photocoagulation and surgery (Bakthavatchalam et al., 2018; Liew et al., 2015). Triamcinolone acetonide (TA) is a kind of synthetic long-acting steroid and has been widely used in the treatment of ME because of its pharmacological actions of anti-inflammation, immune modulation, BRB stabilization and VEGF downregulation (Ip et al., 2004; Moldow et al., 1998). Intravitreal injection and sub-Tenon’s capsule injection are two major approaches administrating TA in ocular tissue. Although intravitreal injection of TA has the advantage of directly loading drug to the target position, multiple secondary complications including elevated intraocular pressure, cataract, vitreous hemorrhage, endophthalmitis have been reported. Delivering TA by a sub-Tenon’s capsule injection is a relatively safe approach for treating ME and has been applied in different retinal diseases (Koga et al., 2005). More recently, Sub-Tenon’s capsule administration of TA has been reported to alleviate RP-ME in a short term (Karasu, 2020). But the long-term effect and visual function outcomes are still unknown. The sub-Tenon’s capsule injection is able to keep and restrict drugs in the sub-Tenon’s capsule space for a relatively long time without allowing diffusion into the orbit tissue, which makes more drugs permeate into the choroid and retina. To treat ME more precisely, we modified the sub-Tenon’s capsule injection technique by replacing the traditional short, sharp needle with a long, curved, blunt needle that is able to run along the surface of the sclera and reach the posterior pole of the eyeball, where the placement of the needle is accurately correspond to the ME lesion. Delivery of TA by a modified sub-Tenon’s capsule injection can facilitate the drug diffusion while avoiding risks and complications secondary to intraocular administration.

Our results suggest administration of TA by a modified sub-Tenon’s capsule injection significantly reduces CMT from 1 week to 2 months following delivery. This sustaining therapeutic effect of TA can be explained by its pharmacokinetics. Former studies have found the vitreous concentration of TA peaked at 12–24 h after sub-Tenon’s capsule injection, and then the drug can be intraocularly present for 8 weeks and longer which is possibly attributed to the binding of TA to retinal pigment epitheliums (Kovacs et al., 2012). The serum concentration of TA has also been tested, and is much lower (tens of nanogram per ml) than that of vitreous body (Kovacs et al., 2012). In spite of the minimal concentration of TA in serum, it can still disrupt the metabolic equilibrium of specific patients such as patients with diabetes (Zaka-ur-Rab et al., 2009; Kovacs et al., 2012). In our study, there were neither definite metabolic abnormalities nor any ocular local side effects observed during all follow ups indicating this modified sub-Tenon’s capsule injection of TA has good systemic and local tolerance.

Given the wide range of biological effects, such as anti-inflammation, immune modulation and neurotrophy, UCMSCs have been applied in many systemic diseases and have been shown to have promising therapeutic effects (Bartolucci et al., 2017; Mukai et al., 2018; Riordan et al., 2018). As to retinal pigmentosa, biological therapy has been long explored (Satarian et al., 2017; Smith, 2004; Zhang, 2016). Recently, the sub-Tenon’s capsule administration of UCMSC has been applied in RP patients. After 12 months follow up, the outcome is significantly beneficial with increased outer retinal thickness and improved retinal function (Ozmert and Arslan, 2020). In our previous study, we found intravenous administration of USMSCs can also stabilize or enhance the overall visual function of RP patents and more notably, can significantly improve their vision related life quality (Zhao et al., 2020). However, it has not been reported if UCMSCs can help relieve ME, a major pathological condition secondary to RP.

In this study, we found intravenous infusion of UCMSCs exerted significant effect on reducing CMT at first month. At 3rd and sixth month, the CMT was still controlled lower than baseline. The long-term acting of USMSCs may be due to their multiple biological effects. In animal models of RP, intravenously administered UCMSCs were found to produce large amounts of neurotrophic factors, and therefore, the photoreceptors were partially protected from apoptosis (Ding et al., 2017; Ng et al., 2014; Wang et al., 2010). Additionally, the infused UCMSCs can directionally migrate to retinal lesions and exert their biological effects to promote the growth of blood vessels, improve the function of BRB and help reconstruct normal retinal structure (Hou et al., 2010; Shibata et al., 2008). The high potential of proliferation and differentiation may make USMSCs keep functioning in a long term when administrated intravenously, which also has been proved by applications in other systemic disease (Bartolucci et al., 2017; Wang et al., 2019). Due to the low immunogenicity of UCMSCs, few adverse effects have been reported after intravenous infusion. A major concern is the change of relevant inflammatory markers. In our study, there were several patients whose IL-6 level were remarkably increased at 1 week after infusion, and dropped down to baseline level subsequently. But the all patients showed no definite clinical symptoms and other severe systemic adverse effects. The increase of IL-6 has also been found in animal experiments reported formerly. Significantly increased IL-6 level was found in cynomolgus monkeys shortly after intravenous infusion of UCMSCs, but recovered in approximately 1 month, and there were no obvious pathological changes associated with the infusion of cells in the general and microscopic examinations (He et al., 2017).

We compared the effects of these two agents in alleviating ME. In TA injection group, patients showed a more rapid reduction of the CMT in the first 2 months and then the macular thickness rebounded to the baseline level, which implied the quick but relatively short-term effect of the TA injection in relieving ME. Whereas in UCMSCs infusion group, the onset of CMT reduction was slower than TA group, but the ME can be continuously controlled till sixth month when the rate of reduction is significantly higher than TA group, which indicated a slow but more persistent action of the UCMSCs. The BCVA and visual field sensitivity of all of the patients in the two groups did not change significantly, and there was no significant difference between the two groups, implying a limitedly improved but stabilized photoreceptor function occurred following the relief of ME. FVEP is used for evaluating the visual function from retinal ganglion cells to the visual cortex. For advanced RP patients, FVEP can still be recordable when the electroretinogram is extinguished. The change of FVEP may be difficult to interpret when visual acuity changes subtly. But, considering the detection is objective and independent of patient’s cooperation, FVEP can still be an important reference for assessing visual function. We performed multiple detections (up to 64 times) to obtain a stable overlayed waveform of FVEP. In our previous study, we found FVEP can be used to evaluate the residual visual function of advanced RP patients (M. Zhang et al., 2021). In this study, the comparison of FVEP between the two groups is similar with that of ME reduction. With relieving of ME, patients in TA groups showed a relatively rapid improvement of P2 wave amplitude (second month), then it dropped down to baseline level. In UCMSCs group, the amplitude of P2 wave was gradually increased to a significant high level at sixth month, at which point it was significantly higher than that in the TA group. This result implied that the UCMSCs infusion may be more beneficial than the TA injection in terms of improving the overall visual function in a long term, which may be due to the wide range of biological effects and more accessible delivery approach from which more retinal neurons such as ganglion cells, bipolar cells can benefit.

In summary, the modified sub-Tenon’s capsule injection of TA and intravenous infusion of UCMSCs were both safe for RP patients with ME. Our results suggested that TA injection can reduce macular edema more quickly and effectively than UCMSCs infusion in the short term, but the effect of the UCMSCs infusion may be more persistent. UCMSCs infusion is more beneficial for improving the overall visual function in a long term. This study demonstrated that both modified sub-Tenon’s capsule injection of TA and intravenous infusion of UCMSCs are promising therapeutic approaches for patients who have RP-ME. Because of the different acting characteristics, these approaches can be applied separately or jointly depending on the disease condition for patients to achieve maximum benefits. Nevertheless, more controlled cohorts and a larger number of subjects are needed to confirm the results.

## Data Availability

The original contributions presented in the study are included in the article/Supplementary Material, further inquiries can be directed to the corresponding authors.
