# Supplementary material for: Comparative Study of a Modified Sub-Tenon’s Capsule Injection of Triamcinolone Acetonide and the Intravenous Infusion of Umbilical Cord Mesenchymal Stem Cells in Retinitis Pigmentosa Combined With Macular Edema
Source: Front Pharmacol. 2021 Sep 27;12:694225. doi: 10.3389/fphar.2021.694225 (PMC8503560; doi:10.3389/fphar.2021.694225)
Supplement: Supplementary file 1 [file DataSheet1.docx]

**Supplementary Data**

**
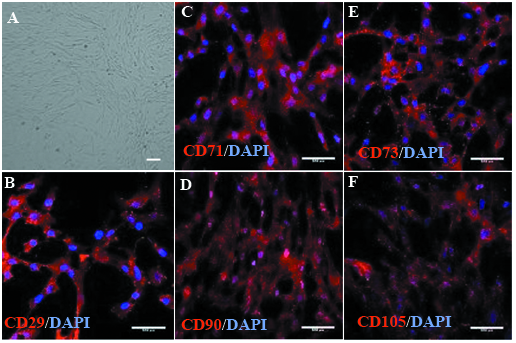
**

**Figure S1 Characterization of UCMSCs.** **(A)** Primarily cultured UMSCs grew adhering to plastic culture vessel. **(B-F)** Immunofluorescence showed positive staining of CD29, 71, 90, 73 and 105. (Scale bar=200μm)

**
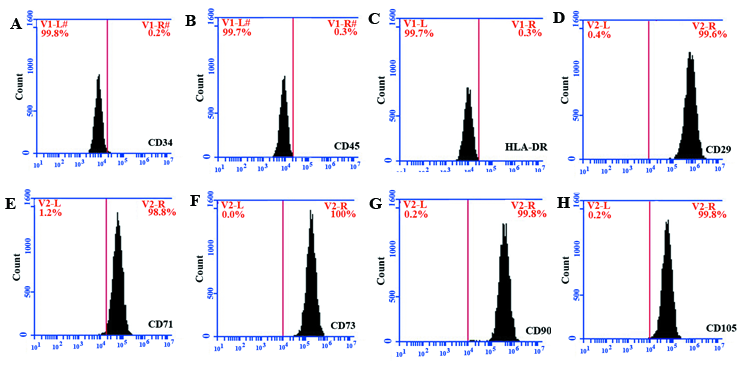
**

**Figure S2 Purity detection of UCMSCs. (A-C )** The flow cytometry showed UCMSCs were negative for CD34, CD45 and HLA-DR (<0.5%), and highly positive for CD29, 71, 73, 90 and 105 (>95%) **(D-H)**.


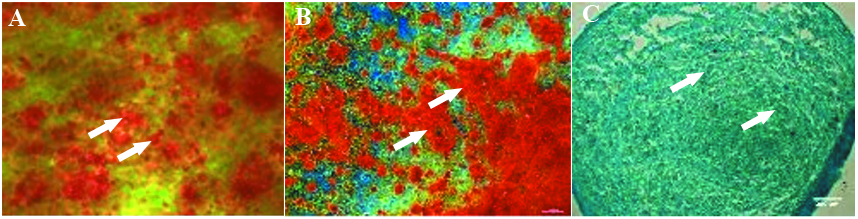


**Figure S3 The Differentiation Test of UCMSCs.** **（A）**Lipogenic differentiation (lipid droplets were shown in red color. White arrows). **(B)** Osteogenic differentiation (calcium nodules were shown in red color. White arrows). **(C)** Chondrogenic differentiation (cell clusters containing acid mucopolysaccharide were shown in blue color. White arrows)

**Table S1 Major blood biochemical parameters**

|  | | Baseline | | | | | 1w | | | | | 1m | | | | | 2m | | | | | 3m | | | | | 6m | | | | |
| --- | --- | --- | --- | --- | --- | --- | --- | --- | --- | --- | --- | --- | --- | --- | --- | --- | --- | --- | --- | --- | --- | --- | --- | --- | --- | --- | --- | --- | --- | --- | --- |
| Subjects | | WBC | ALT | AST | Cr | Glu | WBC | ALT | AST | Cr | Glu | WBC | ALT | AST | Cr | Glu | WBC | ALT | AST | Cr | Glu | WBC | ALT | AST | Cr | Glu | WBC | ALT | AST | Cr | Glu |
| UCMSCs infusion group | UCMSC-P1 | (-) | (-) | (-) | (-) | (-) | (-) | (-) | (-) | (-) | (-) | (-) | (-) | (-) | (-) | (-) | * | (-) | (-) | (-) | (-) | (-) | (-) | (-) | (-) | (-) | (-) | (-) | (-) | (-) | (-) |
|  | UCMSC-P2 | (-) | (-) | (-) | (-) | (-) | (-) | * | (-) | (-) | (-) | (-) | (-) | (-) | (-) | (-) | (-) | (-) | (-) | (-) | (-) | (-) | (-) | (-) | (-) | (-) | * | (-) | (-) | (-) | (-) |
|  | UCMSC-P3 | (-) | (-) | (-) | (-) | (-) | (-) | (-) | (-) | * | * | * | (-) | (-) | (-) | (-) | (-) | (-) | (-) | (-) | (-) | (-) | (-) | (-) | (-) | (-) | (-) | (-) | (-) | (-) | (-) |
|  | UCMSC-P4 | (-) | (-) | (-) | (-) | (-) | (-) | (-) | (-) | (-) | (-) | (-) | (-) | (-) | (-) | (-) | (-) | (-) | (-) | (-) | * | * | (-) | (-) | (-) | (-) | (-) | (-) | (-) | (-) | (-) |
|  | UCMSC-P5 | (-) | (-) | (-) | (-) | (-) | * | (-) | (-) | (-) | (-) | (-) | (-) | (-) | (-) | (-) | (-) | (-) | (-) | (-) | (-) | (-) | (-) | (-) | (-) | (-) | (-) | (-) | (-) | (-) | (-) |
|  | UCMSC-P6 | (-) | (-) | (-) | (-) | (-) | (-) | (-) | (-) | (-) | (-) | (-) | (-) | (-) | (-) | (-) | (-) | (-) | (-) | (-) | (-) | (-) | (-) | (-) | (-) | (-) | (-) | (-) | (-) | (-) | (-) |
|  | UCMSC-P7 | (-) | (-) | (-) | (-) | (-) | * | (-) | (-) | (-) | (-) | (-) | (-) | (-) | (-) | * | (-) | (-) | (-) | (-) | (-) | (-) | * | (-) | (-) | (-) | (-) | (-) | (-) | (-) | (-) |
|  | UCMSC-P8 | (-) | (-) | (-) | (-) | (-) | (-) | (-) | (-) | * | (-) | (-) | (-) | (-) | (-) | (-) | (-) | (-) | (-) | (-) | (-) | (-) | (-) | (-) | (-) | (-) | (-) | (-) | (-) | (-) | (-) |
|  | UCMSC-P9 | (-) | (-) | (-) | (-) | (-) | (-) | (-) | * | (-) | (-) | (-) | (-) | (-) | (-) | (-) | (-) | (-) | (-) | (-) | (-) | (-) | (-) | (-) | (-) | (-) | (-) | (-) | (-) | (-) | (-) |
|  | UCMSC-P10 | (-) | (-) | (-) | (-) | (-) | (-) | (-) | (-) | (-) | (-) | * | (-) | (-) | (-) | (-) | (-) | (-) | (-) | (-) | (-) | (-) | (-) | (-) | (-) | (-) | * | (-) | * | (-) | (-) |
| TA injection group | TA-P1 | (-) | (-) | (-) | (-) | (-) | (-) | (-) | (-) | (-) | (-) | (-) | (-) | (-) | (-) | (-) | * | (-) | (-) | (-) | (-) | (-) | (-) | (-) | * | (-) | (-) | (-) | (-) | (-) | (-) |
|  | TA -P2 | (-) | * | (-) | (-) | (-) | (-) | (-) | (-) | (-) | (-) | (-) | (-) | (-) | (-) | (-) | (-) | (-) | (-) | (-) | (-) | (-) | (-) | (-) | (-) | (-) | (-) | (-) | (-) | (-) | (-) |
|  | TA -P3 | (-) | (-) | (-) | (-) | (-) | (-) | (-) | (-) | (-) | * | (-) | (-) | (-) | (-) | (-) | (-) | (-) | (-) | (-) | (-) | * | (-) | (-) | (-) | (-) | (-) | (-) | (-) | (-) | (-) |
|  | TA -P4 | (-) | (-) | (-) | (-) | (-) | * | (-) | (-) | (-) | (-) | (-) | (-) | * | (-) | (-) | (-) | (-) | (-) | * | (-) | (-) | (-) | (-) | (-) | (-) | (-) | (-) | (-) | (-) | (-) |
|  | TA -P5 | (-) | (-) | (-) | (-) | (-) | (-) | (-) | (-) | (-) | * | (-) | (-) | (-) | (-) | (-) | (-) | (-) | (-) | (-) | (-) | * | (-) | (-) | (-) | (-) | (-) | (-) | (-) | (-) | (-) |
|  | TA -P6 | (-) | (-) | (-) | (-) | (-) | (-) | (-) | (-) | (-) | (-) | * | (-) | (-) | (-) | (-) | (-) | (-) | (-) | (-) | (-) | (-) | (-) | (-) | (-) | (-) | (-) | (-) | (-) | (-) | (-) |
|  | TA -P7 | (-) | (-) | (-) | (-) | (-) | (-) | (-) | * | (-) | (-) | (-) | (-) | (-) | (-) | (-) | (-) | (-) | (-) | (-) | (-) | (-) | * | (-) | (-) | (-) | (-) | (-) | (-) | (-) | * |
|  | TA -P8 | * | (-) | (-) | (-) | (-) | (-) | (-) | (-) | (-) | (-) | (-) | (-) | * | (-) | (-) | (-) | (-) | (-) | (-) | (-) | (-) | (-) | (-) | (-) | * | (-) | (-) | (-) | (-) | (-) |
|  | TA -P9 | (-) | (-) | (-) | (-) | (-) | (-) | (-) | (-) | (-) | (-) | (-) | (-) | (-) | (-) | (-) | (-) | (-) | (-) | (-) | (-) | (-) | (-) | (-) | (-) | (-) | (-) | (-) | (-) | (-) | (-) |
|  | TA -P10 | (-) | (-) | (-) | (-) | (-) | (-) | (-) | (-) | (-) | (-) | (-) | (-) | (-) | (-) | (-) | (-) | (-) | (-) | (-) | (-) | (-) | (-) | (-) | (-) | (-) | (-) | (-) | (-) | (-) | (-) |

ALT: Alanine transaminase; AST: Aspartate transaminase; Cr: creatinine；Glu: glucose

(-): data within normal limits

* : mildly abnormal without clinical significance

**Table S2. Inflammatory Markers Test**

|  | | Baseline | | | 1w | | | 1m | | | 2m | | | 3m | | | 6m | | |
| --- | --- | --- | --- | --- | --- | --- | --- | --- | --- | --- | --- | --- | --- | --- | --- | --- | --- | --- | --- |
| Subjects | | IL-6 | CRP | PCT | IL-6 | CRP | PCT | IL-6 | CRP | PCT | IL-6 | CRP | PCT | IL-6 | CRP | PCT | IL-6 | CRP | PCT |
| UCMSCs infusion group | UCMSC-P1 | (-) | (-) | (-) | 541 | (-) | (-) | 143 | (-) | (-) | (-) | (-) | (-) | (-) | (-) | (-) | (-) | (-) | (-) |
|  | UCMSC-P2 | (-) | (-) | (-) | 425 | * | (-) | 97 | (-) | (-) | (-) | (-) | (-) | (-) | (-) | (-) | (-) | (-) | (-) |
|  | UCMSC-P3 | (-) | (-) | (-) | (-) | (-) | (-) | (-) | (-) | (-) | (-) | (-) | (-) | (-) | (-) | (-) | (-) | (-) | (-) |
|  | UCMSC-P4 | (-) | (-) | (-) | (-) | * | (-) | (-) | (-) | (-) | (-) | (-) | (-) | (-) | (-) | (-) | (-) | (-) | (-) |
|  | UCMSC-P5 | (-) | (-) | (-) | * | (-) | (-) | (-) | (-) | (-) | * | (-) | (-) | (-) | (-) | (-) | (-) | (-) | (-) |
|  | UCMSC-P6 | (-) | (-) | (-) | (-) | (-) | (-) | (-) | (-) | (-) | (-) | (-) | (-) | (-) | (-) | (-) | (-) | (-) | (-) |
|  | UCMSC-P7 | (-) | (-) | (-) | * | (-) | (-) | (-) | (-) | (-) | * | (-) | (-) | (-) | (-) | (-) | (-) | (-) | (-) |
|  | UCMSC-P8 | (-) | (-) | (-) | (-) | * | (-) | (-) | (-) | (-) | (-) | (-) | (-) | (-) | (-) | (-) | (-) | (-) | (-) |
|  | UCMSC-P9 | (-) | (-) | (-) | 325 | (-) | (-) | 112 | (-) | (-) | (-) | (-) | (-) | (-) | (-) | (-) | (-) | (-) | (-) |
|  | UCMSC-P10 | (-) | (-) | (-) | (-) | (-) | (-) | (-) | (-) | (-) | (-) | (-) | (-) | (-) | (-) | (-) | (-) | (-) | (-) |
| TA injection group | TA-P1 | (-) | (-) | (-) | (-) | (-) | (-) | (-) | (-) | (-) | (-) | (-) | (-) | (-) | (-) | (-) | (-) | (-) | (-) |
|  | TA -P2 | (-) | (-) | (-) | * | (-) | (-) | (-) | (-) | (-) | (-) | (-) | (-) | (-) | (-) | (-) | (-) | (-) | (-) |
|  | TA -P3 | (-) | (-) | (-) | * | (-) | (-) | (-) | (-) | (-) | (-) | (-) | (-) | (-) | (-) | (-) | (-) | (-) | (-) |
|  | TA -P4 | (-) | (-) | (-) | (-) | (-) | (-) | (-) | (-) | (-) | * | (-) | (-) | (-) | (-) | (-) | (-) | (-) | (-) |
|  | TA -P5 | (-) | (-) | (-) | (-) | * | (-) | * | (-) | (-) | (-) | (-) | (-) | (-) | (-) | (-) | (-) | (-) | (-) |
|  | TA -P6 | (-) | (-) | (-) | * | (-) | (-) | (-) | (-) | (-) | (-) | (-) | (-) | (-) | (-) | (-) | (-) | (-) | (-) |
|  | TA -P7 | (-) | (-) | (-) | (-) | * | (-) | (-) | (-) | (-) | (-) | (-) | (-) | (-) | (-) | (-) | (-) | (-) | (-) |
|  | TA -P8 | (-) | (-) | (-) | (-) | (-) | (-) | * | (-) | (-) | (-) | (-) | (-) | (-) | (-) | (-) | (-) | (-) | (-) |
|  | TA -P9 | (-) | (-) | (-) | (-) | * | (-) | (-) | (-) | (-) | (-) | (-) | (-) | (-) | (-) | (-) | (-) | (-) | (-) |
|  | TA -P10 | (-) | (-) | (-) | * | (-) | (-) | (-) | * | (-) | (-) | (-) | (-) | (-) | (-) | (-) | (-) | (-) | (-) |

IL-6: interleukin-6; CRP: C-reactive protein; PCT: procalcitonin

(-): data within normal limits

* : mildly abnormal without clinical significance
